# Retraction Note: A novel IgY-Aptamer hybrid system for cost-effective detection of SEB and its evaluation on food and clinical samples

**DOI:** 10.1038/s41598-022-15433-x

**Published:** 2022-06-29

**Authors:** Venkataramana Mudili, Shivakiran S. Makam, Naveen Sundararaj, Chandranayaka Siddaiah, Vijai Kumar Gupta, Putcha V. Lakshmana Rao

**Affiliations:** 1 DRDO-BU-CLS, Bharathiar University Campus, Coimbatore, Tamil Nadu 641046 India; 2 DOS in Biotechnology, University of Mysore, Manasagangotri, Mysore, India; 3 Molecular Glyco-biotechnology Group, Discipline of Biochemistry, School of Natural Sciences, National University of Ireland Galway, Galway, Ireland

Retraction of:* Scientific Reports* 10.1038/srep15151, published online 19 October 2015

The Editors have retracted this Article.

After publication, concerns were raised that the top and bottom panels of Figure 3A appear to be identical. The Authors are unable to provide the original data. The Editors therefore no longer have confidence in the results and conclusions presented in this Article.

None of the Authors has responded to correspondence from the Editors about this retraction.

